# Comparing patient and healthcare worker experiences during a dengue outbreak in Singapore: understanding the patient journey and the introduction of a point-of-care test (POCT) toward better care delivery

**DOI:** 10.1186/s12879-017-2580-9

**Published:** 2017-07-19

**Authors:** Qinghui Tan, Zoe J-L Hildon, Shweta Singh, Jin Jing, Tun Linn Thein, Richard Coker, Hubertus J. M. Vrijhoef, Yee Sin Leo

**Affiliations:** 1 Saw Swee Hock School of Public Health, National University of Singapore, Tahir Foundation Building, Block MD1, 12 Science Drive 2, Singapore, 117549 Singapore; 2 London School of Hygiene and Tropical Medicine, London, United Kingdom; 3 Institute of Infectious Diseases & Epidemiology, Communicable Disease Centre; Tan Tock Seng Hospital, Moulmein Road, Singapore, 308433 Singapore; 4 Mahidol University, 9th Floor, Satharanaukwisit Bldg, 420/1 Rajwithi Rd, Bangkok, -10400 Thailand; 5 National University Health System, Singapore, Singapore; 6 Department of Patient & Care, Maastricht University Medical Center, Maastricht, Netherlands; 7 Department of Family Medicine, Vrije Universiteit Brussel, Brussels, Belgium; 8 Johns Hopkins Bloomberg School of Public Health, Centre for Communication Programs, 111 Market Place, Suite 310, Baltimore, Maryland 21202 USA; 9 Biological Resource Centre, Agency for Science, Technology and Research (A*Star), Singapore, Singapore

**Keywords:** Dengue, Point-of-care testing, Health personnel, Focus groups, Perception, Model of care delivery

## Abstract

**Background:**

In the aftermath of an upsurge in the number of dengue cases in 2013 and 2014, the SD BIOLINE Dengue Duo rapid diagnostic **P**oint-**o**f-**C**are **T**est (POCT) kit was introduced in Tan Tock Seng Hospital, Singapore in June 2013. It is known that the success of POCT usage is contingent on its implementation within the health system. We evaluated health services delivery and the Dengue Duo rapid diagnostic test kit application in Singapore from healthcare workers’ perspectives and patient experiences of dengue at surge times.

**Methods:**

Focus group discussions were conducted with dengue patients, from before and after the POCT implementation period. In-depth interviews with semi-structured components with healthcare workers were carried out. A patient centred process mapping technique was used for evaluation, which mapped the patient’s journey and was mirrored from the healthcare worker’s perspective.

**Results:**

Patients and healthcare workers confirmed a wide range of symptoms in adults, making it challenging to determine diagnosis. There were multiple routes to help seeking, and no ‘typical patient journey’, with patients either presenting directly to the hospital emergency department, or being referred there by a primary care provider. Patients groups diagnosed before and after POCT implementation expressed some differences between speed of diagnoses and attitudes of doctors, yet shared negative feelings about waiting times and a lack of communication and poor information delivery. However, the POCT did not in its current implementation do much to help waiting times. Healthcare workers expressed that public perceptions of dengue in recent years was a major factor in changing patient management, and that the POCT kit was helpful in improving the speed and accuracy of diagnoses.

**Conclusions:**

Health service delivery for dengue patients in Singapore was overall perceived to be of an acceptable clinical standard, which was enhanced by the introduction of the POCT. However, improvements can be focused on **A**dapting to outbreaks by reducing and rendering **W**aiting experiences more comfortable; **A**dvancing education about symptom recognition, while also **R**ecognising better communication strategies; and **E**xpanding follow-up care options. This is presented as the Dengue AWARE model of care delivery.

## Background

Dengue fever (DF) is an endemic disease throughout the tropical world, and is one of the most significant arthropod-borne diseases in the world at present. The disease is caused by four closely related dengue viruses, with the emergence of a fifth serotype, all of which are transmitted by the *Aedes* mosquitoes, principally *Aedes aegypti*. A recent estimate indicates 390 million dengue infections per year, with 96 million manifesting clinically [1]. 3.9 billion people, in 128 countries, are at risk of infection with dengue viruses [2]. Severe dengue, or dengue hemorrhagic fever, is a potentially deadly complication of the disease and proper medical care is needed to avoid complications and risk of death [3].

In Singapore, DF is hyperendemic, with all four dengue virus serotypes circulating. An upsurge in cases occurred recently, and has been associated with the serotype switch from DENV-2, the predominant serotype circulating in Singapore from 2007 to 2012, to DENV-1 in 2013 [4]. The largest spike in dengue cases ever recorded historically occurred in 2013 (22,170 cases, 50% more than the last peak in 2005 [5]) and 2014 (18,335 cases), with 7 deaths occurring in 2013 [6]. In this study, we will refer to this period of time as the ‘dengue surge’ period, the definition of a surge being ‘a sudden, anticipated or unanticipated escalation in health system demand caused by a disease outbreak’.

Early clinical diagnosis is difficult in adults as signs can be subtle, and there can be little commonality in symptoms [7]. Clinicians traditionally make a presumptive diagnosis of dengue fever based on clinical presentation and full blood count results, whilst some use laboratory diagnostic molecular tests for a definitive diagnosis. However, dengue molecular tests are costly and have long turnaround times, with some requiring batch testing which further prolongs the time taken for results to return. In 2011 a cross-sectional study undertaken in Singapore examined 364 Primary Care Physicians’ (PCP) dengue knowledge, attitudes and practices (KAP). It found that KAPs varied amongst PCPs depending on age and practice setting, and less than half ordered dengue diagnostic tests in suspected cases, demonstrating the need for cheaper and more readily available dengue diagnostic tests [8].

Tan Tock Seng Hospital (TTSH) is the major centre for infectious disease referral in Singapore and treated about 37% of dengue public hospital patients in 2005, [9] with the SD BIOLINE Dengue Duo [10] rapid diagnostic at Point-Of-Care Test (POCT) kit being introduced in June 2013 as a quicker and cheaper alternative for diagnosis of dengue fever. Thereafter, a follow-up study done in 2015 indicated that KAPs of PCPs had improved, including more than 80% of PCPs now utilizing dengue diagnostic tests [11].

There has also been an improvement in the diagnostic capabilities of POCT kits in recent years. Previously, many kits tested either NS1, dengue IgG or IgM separately, but the low sensitivities of each test prompted the utilization of the presence of both antigen and antibodies together for a more accurate diagnosis. The POCT kit discussed in this study utilizes all 3 indicators, and was found in a recent local cohort study of 246 patients to have a sensitivity of 93.9% (95% CI 88.8–96.8%) and a specificity of 92.0% (95% CI 81.2–96.9%) [12].

Much exploratory work on dengue focuses on lower or middle-income settings, and community attitudes toward its prevention and control [13–15]. Existing POC testing implementation research has focused on low-resource settings and has determined POC tests to be most useful in areas where central laboratory testing systems cannot efficiently service, and more useful in clinical outpatient settings in a high-income country [16]. However, some studies have also shown that in economically-pressured settings, usage of seemingly simple rapid diagnostic kits may add on an extra layer of work above routine tasks, and using them effectively might very well need exactly the infrastructure they were designed to substitute [17]. In the context of health systems, there can also be barriers to implementing a POC programme effectively [18].

In a high-income country, the circumstances are different and if correctly used in outpatient settings, they could relieve the burden from lab or tertiary care settings. In Singapore, patients are able to visit either a general practitioner (GP) (private sector), a polyclinic (public sector), or the emergency department (ED) of a hospital (either private or public sector) for a consultation. The number of private sector doctors as compared to public sector doctors in Singapore has stood at an approximate ratio of 1:2 in the last 3 years [19]. Dengue patients who are seen at GPs and polyclinics, on top of those who go directly to the ED, are often funnelled into a hospital setting due to a need for further diagnostics, monitoring or admission.

In Dengue endemic Singapore testing is presented as routine, the main issues being getting the tests out as part of usual test batches, fast and efficiently. POC testing was instituted at TTSH to make diagnoses easily ready in time with other routine standard tests, or when labs were short-staffed. In addition, outbreaks become known by using the ‘red dot’ signalling community systems which helped alert communities to outbreaks, aiding symptom recognition and testing decisions. Since there is no specific cure for Dengue, the disease treatment essentially consists of determining the extent of case management; the decision to admit is based on platelet count and symptoms of severe dengue, but hospital guidelines for this vary as protocols in Singapore are not standardized on admission criteria or thresholds for repeated blood counts. These issues will be further elaborated in the discussion.

In our setting, the POCTs have the potential to help reduce unnecessary care seeking visits for patients, relieve hospital congestion, speed up diagnostics, and to be used in an integrated way with existing diagnostics systems and care delivery to improve patient experiences and HCWs workflows. We used a Process Mapping, patient-centred care approach for evaluation. This study aimed to explore dengue surge experiences before and after POCT kit introduction so as to elicit recommendations to improve quality of care delivery for dengue patients in Singapore and in similar high-income endemic settings.

The objectives were to:

1. Unpack how the patient dengue journey (symptom experiences and help-seeking, receiving diagnosis, treatment and recovery) was experienced during a surge in settings that use/do not use the POCT kit.
2. Explore healthcare workers’ (HCW) perspectives and experiences of surge management for dengue (patient arrival, giving diagnoses, treatment and discharge) and the usage of the POCT kit.
3. Synthesise the themes emergent from patient and healthcare workers’ experiences into an improved model of care delivery.

## Methods

### Design

Focus group discussions (FGDs) were conducted with patients groups treated at TTSH before and after POCT introduction; these were anchored around patients sharing their journeys starting from initial symptoms, to diagnosis, treatment and recovery. For HCWs, we undertook In-depth Interviews (IDIs), containing both narratives and semi-structured components. HCWs were of varying seniorities, with some working in Singapore prior to and some after the POCT introduction. Their narratives were elicited around memorable dengue experiences. We describe these methods following the consolidated criteria for reporting qualitative research (COREQ) [20].

### Research team and reflexivity

#### Personal characteristics

Data were collected by four female researchers, trained in qualitative methods. The team was led by ZH an Assistant Professor in qualitative and mixed methods social research, who oversaw data collection. ZH piloted and co-facilitated the IDIs with all team members and lead the FGD work until handing over to QT and SS. A debrief and feedback session was conducted immediately after each FGD to discuss major recurring themes and techniques to improve following FGDs. QT led the analyses supported by ZH.

### Study design

#### Methodological orientation and theory

We aligned our work to a patient-centred care approach [21]. Process Mapping enables the reconfiguring of the patient journey from the patient’s perspective in order to improve quality of care and release resources, and this is centred around the patient’s perspective to identify problems and suggest improvements. We adapted this approach into a framework shaped for our context and study objectives, which operationalized the patient experience as follows: symptom experiences and help-seeking, receiving diagnosis, treatment and recovery. This pathway was mirrored from the clinician’s side as: patient arrival, giving diagnoses, treatment and discharge. These steps were explored with a specific focus on the role of POCT in improving the patient experiences, but we also sought to uncover other factors that were crucial to the patient journey.

During the patient focus groups we encouraged each patient to recount their help-seeking journey, and how the surge context was experienced. This narrative approach generated rich individual level data, which led to participants comparing their experiences among themselves and highlighting similarities and differences. For HCW interviews we followed the interpretive descriptive tradition. Accordingly, we elicited interpretive accounts on the basis of informed questioning, in order to generate knowledge relevant for the clinical context [22, 23].

### Participant selection and setting

**Fig. 1:**
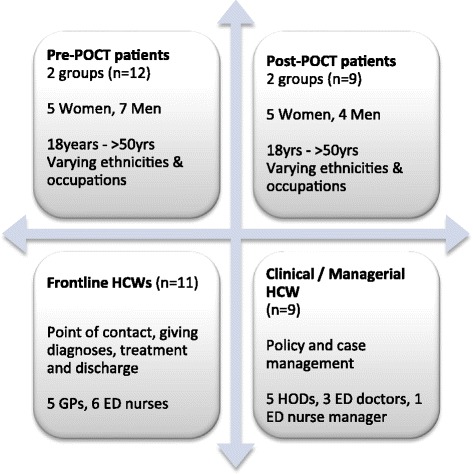
Maximum variation sampling frame

Patients and HCWs were sampled for maximum variation of patient experiences and professional cadres, see Fig. 1. The patients selected and invited were cases with confirmed dengue using RT-PCR or NS1, or cases with compatible clinical diagnosis supported by positive dengue serology. They were a cross-section of age, ethnicity and gender, and from before and after POCT introduction. A variety of HCWs were also sought, from frontline to senior clinical and managerial personnel*.*

### Patients

Participants were recruited through the TTSH laboratory patient database from the years 2012–2014. They were approached via a postal mail invite, *n* = 370 mailers were sent out in total, and non-responders were contacted by telephone. A total of 21 participants (*n* = 10 women, *n* = 11 men) were included in the four focus groups. These represented a range of varying ages, ethnic groups and occupations (Table 1). Two FGDs were conducted with patients who were diagnosed with dengue during the surge period before POCT (June 2013) was introduced, and two with those who were diagnosed after the POCT period. The focus groups were conducted in a meeting room at a centrally located community centre in Singapore. Refreshments and an inconvenience allowance were given to the participants as a thank you gesture.

**Table 1:** Patient socio-demographic characteristics

FGD Group	Gender	Ethnicities	Age groups
Post-POCT 1	3 M, 3 W	2 Chinese, 3 Malays, 1 Bangladeshi	3 younger, 3 middle
Post-POCT 2	1 M, 2 W	2 Chinese, 1 Malay	2 younger, 1 older
Pre-POCT 1	2 M, 4 W	4 Chinese, 2 Indian	1 younger, 4 middle, 1 older
Pre-POCT 2	5 M, 1 W	3 Chinese, 3 Indian	1 middle, 5 older

*FGD* Focus Group Discussions, *POCT* Point of Care Test, *M* Man, *W* Woman

Age groups: younger is ≤30 yrs. old; middle is 31–50 yrs. old; older is ≥51 yrs. old

### Healthcare workers

HCWs were selected so as to cover experiences on all aspects of care – ranging from point of contact, treatment, recovery, case management and policies (Table 2). The hospital HCWs were recruited via snowballing and outreach either through email or face to face; GPs were recruited during the 5th ASEAN Dengue Day Seminar held on 15th June’ 2015 in Singapore. Interviews were conducted in hospital or hotel meeting rooms.

**Table 2:** Healthcare worker characteristics

Group	Gender	Position	Years of experience
Frontline (F)	6 M, 5 W	5 GPs, 6 ED nurses	4 x ≤ 10 yrs
			2 × 11-30 yrs
			5 x ≥ 31 yrs
Clinical care (C)	1 M, 2 W	3 ED doctors	2 x ≤ 10 yrs
			1 × 11-30 yrs
Managerial (M)	2 M, 4 W	5 HODs, 1 ED nurse	1 x ≤ 10 yrs
			3 × 11-30 yrs
			2 x ≥ 30 yrs

*M* Man, *W* Woman, *GP* General Practitioner, *ED* Emergency Department, *HOD* Head of Department

### Data collection

Topic guides were piloted at the outset of data collection; the HCWs’ one prior to fieldwork, and it was also updated inductively as the interviews unfolded. The patients’ guide was tested during the first focus group, although no updates were considered necessary. Consent to participate and for the discussions to be audiotaped was obtained before the start each FGD or interview. The FGDs lasted between 75–90 min whilst the interviews lasted 25–90 min.

### Data analysis

All audio recordings were transcribed verbatim. TQ and SS analysed the data, using a framework approach, following Ritchie and Lewis [24]. This method includes the following steps: 1) familiarisation and confirming the deductive framework; 2) indexing the transcript data to the framework; 3) charting the data in organised columns for analysis; 4) mapping the data, or data analysis, in our case constantly comparing [25] for first and second order themes; and interpretation. NVivo 10.0 and MS Word 2007 were used to manage data, and to help the charting process. Thematic first order analyses were carried out for patients according to the framework topics as follows: 1.1 symptom experiences and seeking help; 1.2 getting diagnosed; 1.3 treatment and recovery. And, for HCWs frameworks were analysed as follows: 2.1 patient arrival to care; 2.2 giving diagnoses; 2.3 treatment and discharge. Second order analysis contrasts patient and HCW themes; interpretation related these findings to a model of care delivery.

## Results

**Fig. 2:**
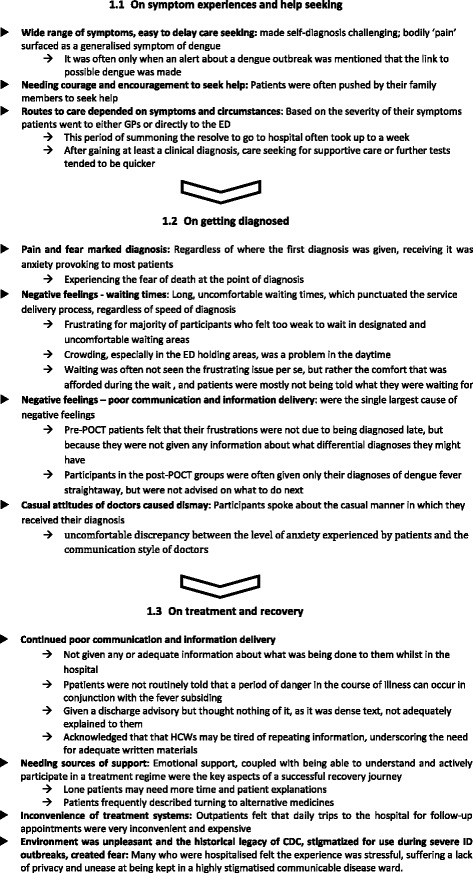
Themes describing patient experiences

**Fig. 3:**
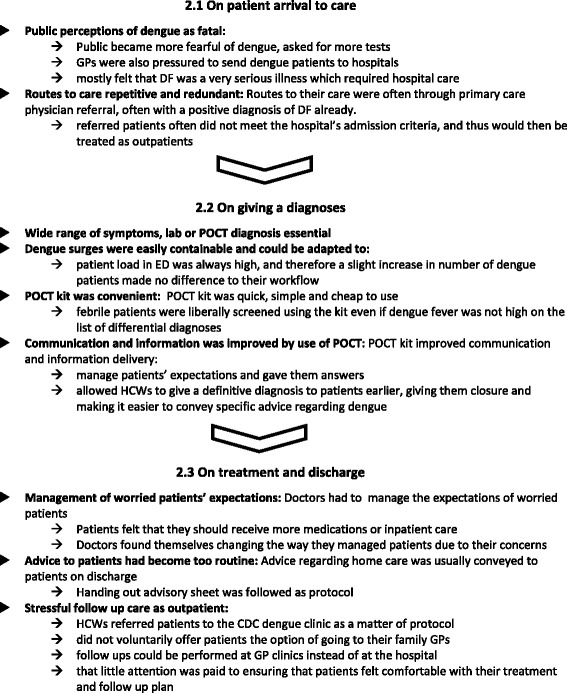
Themes describing health care workers experiences

**Fig. 4:**
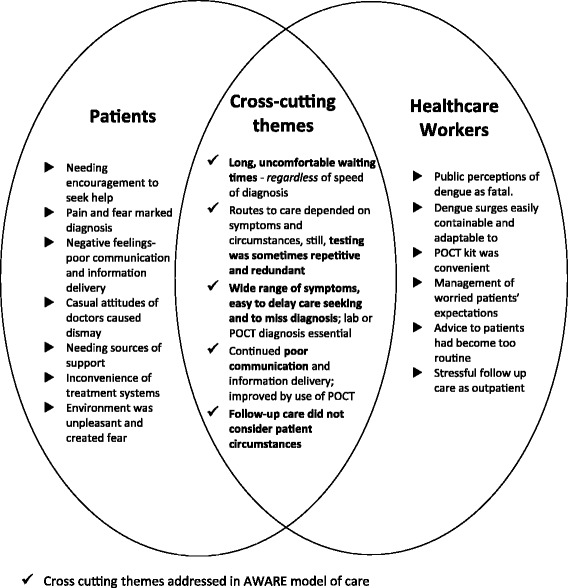
Summary of key and overlapping themes from patients and healthcare workers

**Fig. 5:**
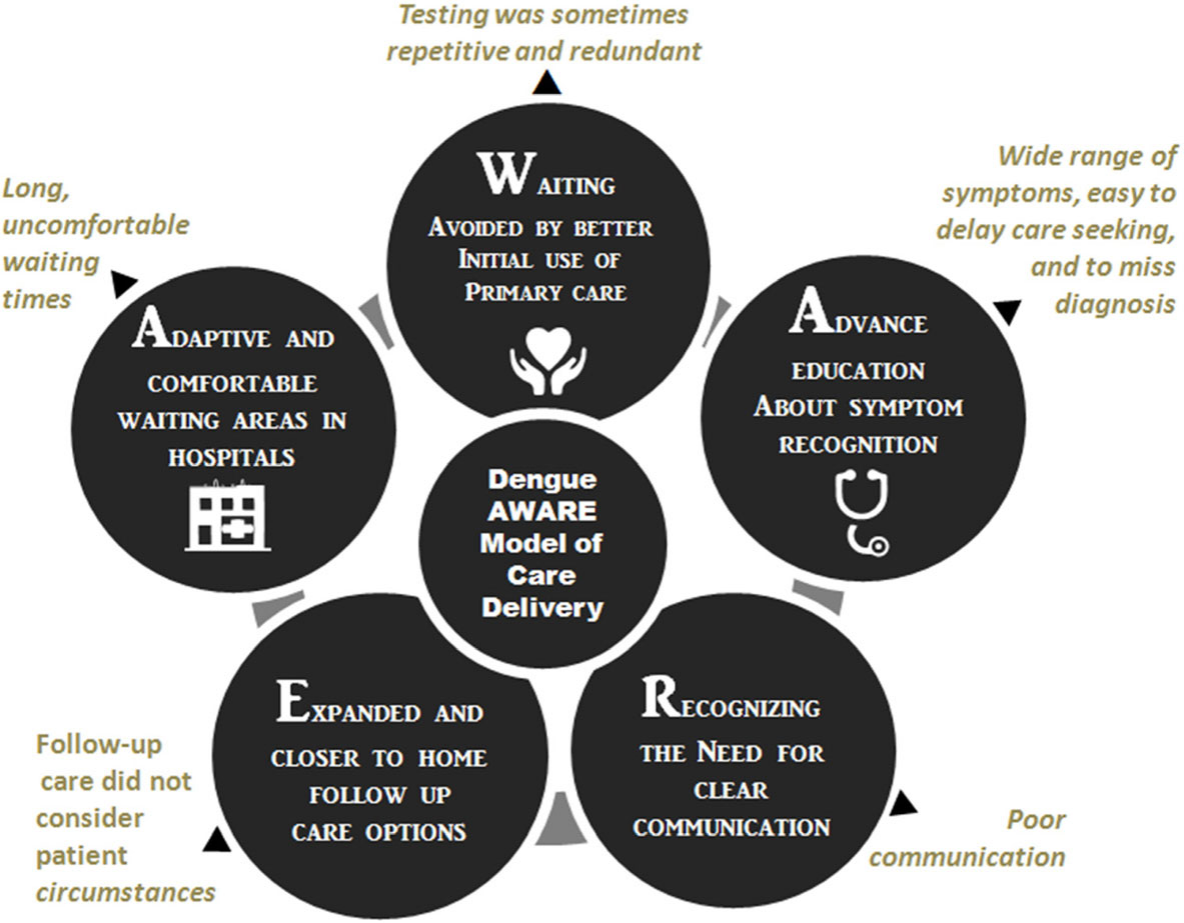
Shared patients and healthcare workers’ themes driving Dengue AWARE model of care delivery

The first order themes are reported in *italics*. The HCW themes are compared across the patient ones summarised in Figs. 2 and 3. Second order themes are analysed by combining patient and HCW data, see Fig. 4, these are synthesised into a new theoretical set of constructs and then pragmatically interpreted into model of care, in Fig. 5.

## Findings for patients

### On symptom experiences and help seeking

#### Wide range of symptoms, easy to delay care seeking

*S*ymptoms were reported to be very varied, ranging from a combination of high fevers, headaches, fainting, weakness, rash and itching to simply feeling very tired. *Such a wide range of symptoms made self-diagnosis challenging*. Fever, the main known symptom of dengue was not always present: “case is, I don’t have fever [surprised sighs from the group], I don’t know why! I only got the headache, the severe headache, the probing headache” [pre-POCT 2, M, Chinese, older]. As another participant put it: “so I knew something was wrong, but I didn’t think I had dengue. I actually thought maybe I was just tired… because I’m anaemic also, so there’s the thing that I’m always tired” [post-POCT, W, Malay, middle]. It was surprisingly common for patients’ symptoms to be so non-descript that they just put them down to existing health issues.

#### Needing courage and encouragement to seek help

Despite having fevers, some participants reported trying to carry on with normal routines for a few days, not suspecting dengue, thinking the fever would pass quickly: “that period of time is like you know something is going on you’re not normal […] it’s only a fever, but it didn’t accompany with flu, or cough” [pre-POCT, W, Indian, middle]. Due to the wide range of systems, *bodily ‘pain’ surfaced as a generalised symptom of dengue* although, still, most participants reported this in varying degrees, from mild malaise, to aching joints: **“**very pain lah! I sleep also cannot. Standing also cannot.” [Post-POCT 1, M, Indian, middle], to bone-breaking sensations: “the word I think of is crackling because it feels like your bone is crackling so… yeah, it kind of hurts” [post-POCT 1, M, Chinese, younger].

As even this commonality was varied – *it was often only when an alert about a dengue outbreak was mentioned that the link to possible dengue was made*. One participant described: “No I never think of it [as dengue]. Then my neighbour said, ‘oh downstairs [someone] got dengue.’ Because I don’t feel any bite or anything, I don’t know why all of a sudden the temperature go very high.” [pre-POCT 2, M, Chinese, older]. Sometimes this alert came from a community alert system that features a red ‘dot’ signal by the side of the road to warn about a dengue outbreak [26], as one person put it: “So then, I saw the banners in my area: red. The red dot thing [dengue alert]. The banner had. Hmm, maybe I have that! I was like, I don’t have any other symptoms and all [except being tired], I don’t have this [or that]: I have no rashes nothing, no fever nothing. Then my husband said maybe you should just go and check it out…. So I went to my GP” [post-POCT 1, W, Malay, middle]. *Patients were often pushed by their family members to seek help*, or ‘take the courage’ to go to the ED.

#### Routes to care depended on symptoms and circumstances

*Based on the severity of their symptoms patients went to either GPs or directly to the ED*, with less severe cases going to GPs first, “My mom is the one who encouraged me to go and see a doctor. Once I went to the polyclinic (name), they told me it’s just a normal fever. But then after five days of not recovering, I took the courage to go to the Tan Tock Seng” [post-POCT 2, W, Malay, younger]. *This period of summoning the resolve to go to hospital often took up to a week*; however when people had trusted a convenient primary care giver (e.g. GPs) nearby *gaining them at least a clinical diagnosis, care seeking for supportive care or further tests tended to be quicker*. For example: “I’ve got a family doctor and she’s always very frank. She says you probably have dengue; they wouldn’t be able to do anything about it, but if it gets too bad, go in because you’re going to have to start doing your daily blood tests. So I think I, I check myself into A&E Tan Tock Seng, about two days, two, three days after I got my first symptoms” [Post-POCT 2, M, Chinese, younger].

In sum, participants reported a variety of routes to getting medical attention. Some with milder symptoms went at first to their GPs and polyclinics, before being referred or getting sicker and going to the hospital. It was not uncommon to go straight to the ED, but often prompting was needed to provoke the suspicion of dengue, or encouragement to face diagnosis.

### On getting diagnosed

While the Dengue Duo POCT was sometimes used by GPs, this diagnosis nevertheless entailed further lab work that most outpatient clinics did not have, leaving it to tertiary care centres to carry out platelet counts and assess disease severity. Getting diagnosed at a GP clinic therefore did *not* work to reduce ED congestion; it simply added another step to bringing patients there eventually. *Regardless of where the first diagnosis was given, receiving it was anxiety provoking to most patients*.

#### Pain and fear marked diagnosis

Many participants talked about *experiencing the fear of death at the point of diagnosis*, as well as throughout the course of the illness. Some were even resistant about confirming their diagnosis, because of a highly publicized death of a young man in 2013 [29], and it was commonly expressed: “people die, people die I scared already” [Post-POCT 1, M, Bangladeshi, middle]. Feelings of having survived a traumatic event – apprehension, frustration, anger, confusion, were mentioned frequently during the discussions on their dengue journey. Thus, *harbouring negative emotions* were expressed by almost all participants, regardless of whether they were from pre or post-POCT groups at various points, due to bottlenecks which will be discussed next.

#### Negative feelings- waiting times

One bottleneck was *long, uncomfortable, waiting times, which punctuated the service delivery process, regardless of speed of diagnosis*, as most participants spoke about an average waiting time in the ED of 3–4 h, before being seen by a doctor. This was added upon by 2–3 h of waiting for blood test results, and a further few hours to have a medicine prescription and next steps: **“**I had to wait three hours for a prescription. So I got quite frustrated.” [post-POCT 1, M, Malay, middle]. This long process made it very *frustrating for the majority of participants who felt too weak to wait in designated and uncomfortable waiting areas. Crowding, especially in the ED holding areas, was a problem in the daytime*. For patients who consulted past midnight, crowding was not an issue; patients who were able to gain a bed while waiting were less upset about delays. It emerged specifically that *waiting was not often seen as the frustrating issue* per se*, but rather the comfort that was afforded during the wait*, and the fact that *patients were mostly not being told what they were waiting for*.

#### Negative feelings – Poor communication and information delivery

Bottlenecks around *communication and information delivery were the single largest cause of negative feelings* for most participants. This theme straddles both the topic of getting diagnosed, and our following section on treatment and recovery. Some participants in the pre-POCT groups were only told that they had dengue fever after going home or after a few days of being hospitalized. However, *pre-POCT patients felt that their frustrations were not due to being diagnosed late, but because they were not given any information about what differential diagnoses they might have*, nor about what they were being treated for. In contrast, *participants in the post-POCT groups were often given only their diagnoses of dengue fever straightaway, but were not advised on what to do next*. As one person put it: “They didn’t tell us what to look out for. Just tell us like okay you have dengue, just go home. That’s it, we know that now we are a statistic.” [Post-POCT 1, W, Malay, middle].

#### Casual attitudes of doctors caused dismay

*Participants spoke about the casual manner in which they received their diagnosis*: “My body hurts, and this guy is looking at me just smiling, yeah you have dengue, like so, yeah I didn’t know how to put the whole situation together.” [Post-POCT 1, M, Malay, younger]. Overall, we observed an *uncomfortable discrepancy between the level of anxiety experienced by patients and the communication style of doctors*; this was regardless of speed of delivery of diagnosis, which indeed appeared greatly improved by uptake of the POCT. Yet, this advance was overshadowed by an insensitive bedside manner, other persistent reasons for prolonged waiting regardless, in largely uncomfortable seating areas.

### On treatment and recovery

#### Continued poor communication and information delivery

Almost all participants, both outpatient and hospitalized, voiced that they were *not given any or adequate information about what was being done to them whilst in the hospital*, e.g. why their blood was being drawn daily, what medications they were given, and what to do at home. As one inpatient stated, “I was really really weak, and I had no energy to kind of ask them what’s happening you know? Why is it taking so long, I had really no energy. And I was so low, I was angry, I was frustrated, I was depressed and everything was there.” [Pre-POCT 1, W, Indian, middle]. Patients were often not privy to results of blood tests even on asking. Given the nature of blood-related illnesses, natural anxieties arose about having somehow acquired a potentially chronic disease such as HIV or Hepatitis C, which made not being given results of repeated blood tests more stressful.

*Participants were not routinely told that a period of danger in the course of illness can occur in conjunction with the fever subsiding*, **“**So, I was also only after I went to (another hospital) I got to know that it’s only when the fever subsides, that’s the dangerous period. So I was like, so you mean there’s more?” [Post-POCT 1, M, Malay, middle]. Some described being *given a discharge advisory but thought nothing of it, as it* was *dense text, not adequately explained to them*. It was also *acknowledged that HCWs may be tired of repeating information*, **“**Because I feel that the nurses there ah, they are already very tired of explaining you know. If everybody asks they will be so tired” [Pre-POCT 2, W, Chinese, older], u*nderscoring the need for adequate written materials*.

With regards to knowledge and information seeking on dengue, many participants described receiving an abundance of preventative information. The ‘Do the Mozzie Wipeout’ campaign [28] in Singapore was so effective that they all knew about the steps it took to prevent dengue, but not what to do after they had contracted it. *Knowledge of dengue boiled down to* one commonly stated theme: ***“*** *The only thing I know about dengue is how to prevent dengue”* [Post-POCT 1, M, Malay, younger]. Most participants who did not receive adequate answers from HCWs about how to manage their conditions turned to the internet for information, though they understood that the internet may not give the best opinion for their case. Some participants who were admitted used their time in hospital to search for information on their smartphones, but noted that it was tiring to have to be seeking information themselves on a small screen, whilst they were ill. One, minority feeling, was that it was down to the individual to educate themselves – but *most people said simple, step-by-step information on how to manage dengue and the reasons for different treatment initiatives would have helped them to cope better*.

#### Needing sources of support

*Emotional support, coupled with being able to understand and actively participate in a treatment regime were the key aspects of a successful recovery journey*. Most participants received support from family members, **“**For me my whole experience I think, erm was made bearable because of the morale support” [pre-POCT 1, W, Chinese, younger]. A transient worker, alone in Singapore, was hospitalised the longest, and described having no one to push doctors for answers on her behalf, and being too weak to absorb things easily: **“**And yes [about getting] the medical [feedback]… at least if you have some person to stand by you, you know. For me my case definitely I needed it the most… because there wasn’t anybody” [pre-POCT 1, W, Indian, middle]. *Lone patients may need more time and patient explanations*. Due to the ambiguity of the disease treatment plan, which requires disease management rather than cure, *patients frequently described turning to alternative medicines* – which included drinking water boiled with papaya leaves, and green tea.

#### Inconvenience of treatment systems

After being diagnosed, very few patients were admitted, and most whose platelet counts were below a certain level were asked to return to the Communicable Disease Centre (CDC), situated next to TTSH, for repeated blood counts. Despite there being several guidelines for the treatment of DF in Singapore, there is no standardized admission criteria, nor a standardized threshold for repeated blood counts. Participants treated as *outpatients’ felt that daily trips to the hospital for follow-up appointments were very inconvenient and expensive*, especially taking transport back and forth to the hospital. They were not informed of an option to return to their family GPs or polyclinics for follow-ups. “And yeah, the other frustrating part was is I’m so sick I still need to come to [hospital] every day, and then I have to get myself, erm the get the blood test and then wait two, three hours there…It wasn’t a very comfortable place, and you just had to keep coming back when you just want to sleep at home.” [Pre-POCT 1, W, Chinese, younger].

#### Environment was unpleasant and the historical legacy of CDC, stigmatised for use during severe infectious disease outbreaks, created fear

Participants from both groups, especially those who were hospitalised at the CDC older facility, were very uncomfortable about their environment. They described feeling stigmatized, fearful, unpleasant, and under strict control. A participant described hearing noises at night, and after being told by a HCW that it was frequently heard, started to imagine that the place was haunted. Some thought that they were hospitalised in the CDC because of the ‘bed crunch’ at TTSH, which added on to their misery. In describing the CDC, a participant said it was “Like a death sentence (group laughter)” [Pre-POCT 2, W, Chinese, older] *Many who were hospitalised felt the experience was stressful, suffering a lack of privacy and unease at being kept in a highly stigmatised communicable disease ward.*

### Findings for healthcare workers

GPs interviewed talked about their general experiences with dengue patients during surge periods, whilst hospital HCWs described how they managed dengue patients from point of entry in the ED till the point of discharge, as well as the advantages of the POCT kit.

### On patient arrival to care

#### Public perceptions of dengue as fatal

An over-arching theme that emerged in our discussions with GPs and hospital HCWs was the *public perceptions of dengue* and heightened awareness in 2013, when they saw a spike in the number of dengue cases, and the first death from dengue of a healthy young man, which was widely publicised in the media [27]. The heightened awareness of DF affected the way HCWs had to manage patients throughout their arrival, diagnoses, treatment and recovery. One ED doctor stated, “Yes so, unfortunately because of this, the public *asked for more tests*. As in everybody who comes in for dengue screen wants to know whether they have dengue or no [....] Because of the recent death, everybody actually have to change the way we approach the management of patient with dengue.” [C1]. As the *public became more fearful of dengue*, *GPs were also pressured to send dengue patients to hospitals* either because patients requested to be referred, or because they were not very confident about handling certain cases. Patients *mostly felt that DF was a very serious illness which required hospital care* of some sort, and therefore often felt more comfortable being referred to a hospital. As one senior level official put it, “So there were actually very strong pressures both media wise you know. Just fear factor funding issue” [M1].

#### Routes to care repetitive and redundant

Hospital HCWs acknowledged that patients’ *routes to their care were often through primary care physician referral, often with a positive diagnosis of dengue fever already*. Those who did not have official records of their laboratory or diagnostic tests would then have their tests repeated at the hospital, “Yes Yes. Or the results were positive la. Because I don’t think GP has an idea of, may not have an idea of how we manage.” [F6] However, *referred patients often did not meet the hospital’s admission criteria, and thus would then be treated as outpatients,* defeating the purpose of their referral. “So if they come to you for what? Because you will just tell them to go home.” –Interviewer, “Yes. [Laughter] So I think there might need to be something to close the gap between the primary healthcare and the acute institutions.” [F6] In contrast, a GP who operated out of a clinic in a private hospital commented that they would admit patients as long as they could pay, which highlighted a difference between the management of patients in public and private healthcare.

### On giving a diagnoses

#### Wide range of symptoms, lab or POCT diagnosis essential

Like the patients, HCWs reported that dengue patients normally presented with hard to discern symptoms, no different from other febrile patients, making the testing central to diagnosis*:* “a lot of times they come in with fever and flu symptoms of gastroenteritis.” [C2] Many HCWs expressed difficulties with diagnosing DF based on presenting symptoms, and agreed that a diagnostic test was very helpful in screening for any febrile patient even mildly suspected of having dengue.

#### Dengue surges were easily containable and adaptable to

Despite the surge in dengue cases in 2013 and 2014, almost all ED HCWs reported not feeling an impact. They felt that the *patient load in ED was always high, and therefore a slight increase in number of dengue patients made no difference to their workflow*, especially since its mode of transmission was not airborne, “Hmm... from personal perspective […] the dengue surge was not something that had a great impact on us as a department, emergency department.” [F6] Some GPs said that they did see a few extra cases of dengue, but that it did not affect their workload. All reported that the hospital system was able to cope well. “Actually, I didn't feel that much, you know, day in day out we just work…I don't feel stress because of the dengue patients coming in. Anyway, it is not, they are not you know, spread through, droplets or whatever you know, they are just you know bitten by mosquito you know” [F7].

#### POCT kit was convenient

HCWs felt that in contrast to other methods of dengue diagnoses, the *POCT kit was quick, simple and cheap to use*. The turnaround time was greatly reduced, as tests could be performed when necessary, whereas molecular tests needed to be batched as well as take longer to run. Nurses were also empowered to order the test at triage. As a result, *febrile patients were liberally screened using the kit even if dengue fever was not high on the list of differential diagnoses*. Doctors described that patients with symptoms not typically associated with DF sometimes had positive blood test results, “But there’s been numerous incidences where for some reasons, either myself or someone does the test and it turns out to be positive” [C2], which surprised them many a times.

GPs who used it found it convenient, and they were able to do it at actual point of care, “Yes. So… and like that… if let’s say there’s no patient waiting, I can wait with the patient with the cassette in front of him, if not I tell him to leave the room, and I’ll see him again in 20 minutes time.” [F4]. At TTSH, it was mentioned that usage was easy and that the staff at the existent Methicillin-resistant *Staphylococcus aureus* screening lab were trained to conduct the test. “we already had a staff working in ED, 24*7, we trained them to do this test. It is not a difficult test to do. A bit time consuming, 20 minutes you have to wait for the reading.” [M4] The GPs talked about being able to conduct the test in front of patients and finding it simple to use; complaints were not about the usage of the kit, but the cost, which caused some patients to decline it: “But the downside is that because it costs money…” [F4]. A hospital HCW found that sending blood tubes to the laboratory for testing brought with it problems of tubes getting lost; but generally the rest did not have any complaints about it as it the charges were low.

#### Communication and information was improved by use of POCT

HCWs also felt that the *POCT kit improved communication and information delivery.* They expressed that the quick diagnoses helped to *manage patients’ expectations and gave them answers*, rather than improve case management. This was especially important because of the change in public perceptions of dengue and the increased fear that patients were experiencing regarding their conditions. Positive results *allowed HCWs to give a definitive diagnosis to patients earlier, giving them closure and making it easier to convey specific advice regarding dengue*, “Ok ya definitely. Because with this we can tell the patient, ok you are having dengue fever.” [F7]. They also felt that dengue patient management and outcomes were improved because of the definitive diagnosis. None said that they had any problems with false negatives as patients with symptoms were always advised to look out for signs of deterioration.

### On treatment and discharge

#### Management of worried patients’ expectations

Following the surge, doctors were trying to reduce admission rates as research findings had identified risks associated with progression of the disease and found that most dengue patients could be managed appropriately as outpatients. This was in contrast to patients’ concerns and expectations to be admitted. Treatment was based on clinical criteria and following hospital admission guidelines, rather than simply the diagnosis itself, and as a result, doctors found themselves having to *manage the* expectations of worried patients *who felt that they should receive more medications or inpatient care*. One doctor expressed that even though the POCT kit helped with the diagnosis of dengue, he still *found himself changing the way he managed patients due to their concerns*, rather than their actual conditions, “It is more to do with trying to manage expectation, rather than to change the type of treatment we are treating for dengue. So that was something we had to change.” [C1].

#### Advice to patients had become too routine

HCWs said that some form of *advice regarding home care was usually conveyed to patients on discharge*, although less information was given if they were busy. The discharge advice also often came from the nurses, “The nursing part we usually follow whatever written on the pamphlet to convey to the patient. Like come back if you are unwell. Look out, if you have dengue, look out for signs of bleeding, and that kind of stuff.” [F6] As medications were only symptomatic, the most frequent advice given was to drink plenty of water and to watch out for worsening symptoms. Some would also advise patients regarding alternative treatments like boiled papaya leaves. Doctors would print out an advisory sheet for patients, explaining it once through either briefly or thoroughly, whilst nurses would explain it again at the point of discharge. “We don’t go through line to line for them to read. But sometimes we point out the important things la. Especially like those a bit older, err, come alone, then we make sure that they really know.” [C3] *The handing out of the advisory was followed as a protocol*, and it did not seem like any HCW felt that the advisory was lengthy or unsuitable for the patients.

#### Stressful follow up care as outpatient

It was generally agreed that follow up care was usually on an outpatient basis, and *HCWs also referred patients to the CDC dengue clinic as a matter of protocol*. They *did not voluntarily offer patients the option of going to their family GPs* unless patients asked. At the CDC, patients could be asked to return daily for blood tests till their platelet counts exceeded the stipulated threshold level. Whilst some HCWs did not consider the cost and inconvenience involved for patients who were asked to return to the CDC, some doctors did express that follow ups and blood counts could certainly be performed easily by GPs, “and then but 80% got discharged, we then have to come to our clinic to be followed up daily, whereas actually it’s something that the GPs could do” [M1] It was clear *that follow ups could be performed at GP clinics instead of at the hospital; and little attention was paid to ensuring that patients felt comfortable with their treatment and follow up plan*.

### Comparing findings for patients and healthcare workers

Themes which surfaced in both patient and HCW groups generally indicated that dengue surge preparedness in Singapore for endemic dengue is adequate. In addition, introduction of the POCT kit did help to improve speed and accuracy of diagnoses, but not, as currently implemented, to reduce waiting times. There were individual and shared themes between the patient and HCW groups, with groups differing in views for the majority of shared themes. We aimed to match what patients and HCWs said about their experiences, and reveal what was clearly contrasting in their opinions. Second order overlapping themes are summarised in Fig. 4. There showed clear patterns regarding: Long uncomfortable waiting times; testing sometimes being repetitive and redundant; the wide range of dengue symptoms were problematic, making it easy to delay care seeking and to miss diagnosis; and follow-up care not considering patient circumstances.

These cross-cutting themes were synthesised and presented as the **Dengue AWARE model of care** delivery in Fig. 5. It is recommended that this model of care be taken into consideration to structure planning of health service delivery for dengue patients in over-stretched, and surge-likely dengue endemic settings. Dengue AWARE steps are described in turn below.

- **▶ A**daptive waiting strategies and comfortable waiting areas in hospitals

Waiting times are an enduring problem in the ED worldwide [28, 29]. It is simplistic to recommend reducing waiting times through various means; therefore it is proposed that there could be a practical reinvention of the wait time experience instead. As it was found that patients were contented to have somewhere to rest whilst waiting, reclining chairs at the ED and pharmacy waiting area could be provided for febrile patients to rest or sleep on, together with cushions with disposable covers and blankets. A ‘care corner’ [30] with dedicated staff to provide regular updates to family members will be useful in helping to alleviate the frustrations of patients and family. Mobile charging stations, free wifi and generally making waiting more bearable through these enhancements would help patients remain comfortable until seen.

A ‘wait at home’ and be notified of your appointment 20 min in advance service is also a good option for those in proximity to the hospital. Although the improved speed of diagnosis did not appear to impact waiting times much, reducing ED crowding by better sharing of POCT and other test results, as suggested below, may help.

- **▶** ***W*** *aiting avoided by better initial use of primary care*

Patients at the hospital were often being sent home after referrals from primary care centres for admission, and often managed the same way as they would have been at the primary care centre. There could be standardized admission criteria for DF across all restructured hospitals so that PCPs have a better idea of whether patients are suited for hospitalization. There could also be better coordinated care between primary and tertiary care centres- GPs regularly referring to a particular hospital should have the admission criteria of the hospital, and all healthcare centres should make laboratory and POCT test results readily available on paper to patients to avoid duplication of services.

- **▶ A**dvance education about symptom recognition

Because Patients and HCWs had dissimilar views about the adequacy of communication and information. All inpatients could receive a flyer distributed on admission which describes what they may go through as a hospitalized patient. It should include information about the frequency of blood tests and explanation of what they are for, as well as a description of the symptoms they may experience. As many patients were concerned about the lack of advice regarding the symptom recognition for severe dengue, the usage of mobile phones can be leveraged on. Patients can receive a Short Message Service (SMS) with a hyperlink to the hospital website which could include reliable information regarding dengue aftercare, highlighting warning signs for severe dengue.

- **▶ R**ecognizing the need for clear communication

The existing flyer distributed to patients on discharge at TTSH was plain and wordy, and was not memorable to patients. It should highlight the gaps in knowledge that patients had described, and include simple explanations and illustrations of symptoms and disease progression, warning signs and detection of disease severity, home fever management and supportive treatment for DF symptoms. Adequate simple information would help alleviate patients’ fears. Doctors must be encouraged to briefly describe the flyer to patients during consultation.

‘Trigger films’ [31] are part of the British National Healthcare System project to facilitate quality improvement, whereby patients and their caretakers freely narrate experiences about their conditions and how they felt about the care they had received. By watching ‘trigger films’, HCWs are able to get a better idea of the positive and negative experiences of patients. Training for HCWs and reassurance for patients can be encouraged by the making of similar short clips with dengue patients narrating experiences around the core themes we identified.

- **▶ E**xpand and provide closer to home follow up care options

Outpatients were concerned that daily trips to the hospital were inconvenient, and many appeared unaware they could follow up at primary care centres. To make dengue services more convenient, stable outpatients at hospitals could be advised that they are able to follow-up at a primary care centre closer to them.

## Discussion

Due to the heterogeneity of symptoms, routes to care, case management based on symptom severity and lack of standardised admission and follow-up criteria, there was no ‘typical’ Dengue patient journey. However, opinions that surfaced within patient groups in this study have previously been found in ED patients in general and not limited to only dengue patients. A paper which analysed the complaint rates in the ED of another public hospital in Singapore between 2002 and 2003 found that organization/logistics (waiting time, lack of interim care while awaiting doctor’s review or admission) was the biggest complaint (46%); followed by communication (26%) [32]. Communication also surfaced as a major issue in ED complaints around the world [33]. These were similar to the subthemes of communication/information and waiting times in this study being the largest causes of negative feelings in patients. Therefore, although the recommendations elicited here were targeted at health service delivery for dengue patients, some can be applied more broadly.

In Singapore, the government has implemented a large number of measures to cope with the dengue epidemic. These include increasing subsidies for dengue NS1 testing, investigations into dengue deaths to ensure that clinical management was consistent with accepted standard clinical practice; improving clinical management by sharing good practices; issuing circulars to doctors and hospitals to provide updates on the dengue situation [34]. The latest government guidelines for the management of dengue fever was issued in 2002. Subsequently, the Ministry of Health issues a yearly dengue surveillance report, and circulars to doctors and hospitals to provide updates on the dengue situation and reinforce advice about the clinical management of dengue or suspected dengue patients. Doctors are advised to monitor dengue patients closely, and to look out for warning signs and symptoms which may warrant a referral to hospital for further medical evaluation and management. Hospitals are also reminded to ensure that for suspect or confirmed dengue patients who are clinically assessed to not require admission at that point in time, there are outpatient monitoring systems to review them. In addition, hospitals have been informed that suspect and confirmed cases of dengue who return to emergency departments within 24 to 48 h should be appropriately prioritised at triage [35].

The admission criteria for TTSH from 2007 onwards included the following: platelet count ≤50,000/mm3, serum hematocrit: ≥50%, blood pressure ≤ 90/60 mmHg, postural drop in blood pressure .20 mmHg, pulse ≥100/min, clinical bleeding (except petechiae), clinically unwell patients (in particular, severe abdominal pain, persistent vomiting), elderly patients with comorbidities (such as diabetes, hypertension, heart failure, cancer, stroke), and whether patients fulfilled the TTSH severe dengue predictive model, which included a computerized predictive equation using clinical bleeding, lymphocyte proportion, serum urea and protein levels to generate an output of high vs low risk for severe dengue and a decision tree with three decision nodes of clinical bleeding, urea .4 mmol/l and protein ≤67 g/l. Fulfillment of any one of the eight criteria was deemed appropriate for admission [9]. Post 2009, the presence of several warning signs was also introduced to the criteria list. Patients deemed suitable to be treated as outpatients would be asked to follow-up at the Communicable Diseases Centre (next to TTSH) for full blood counts, generally till their platelet counts are on the upward trend.

As for GPs, the Ministry of Health has recommended that when making referral decisions, platelet count should be interpreted together with significant clinical signs and symptoms, which may include bleeding, change in mental status, abdominal pain, hypotension and narrowed pulse pressure. The challenge for the primary care physician then is to find that delicate balance between sending a patient to hospital unnecessarily and missing a potentially severe case of dengue [35]. Understandably, many GPs do not want to miss a potentially severe case of dengue, especially in the wake of the dengue patient death in 2013, and would refer a patient off to the hospital to be safe rather than sorry. This naturally resulted in a fair proportion of patients being turned down admission if subsequent diagnostic tests at the hospital deemed them fit enough to be treated as outpatients.

Overall, our findings suggest that clinical management of dengue fever is of an acceptable standard, and patients rarely complained about their clinical treatment. Whilst the POCT kit did improve speed and accuracy of diagnoses, it was clear that waiting times, a lack of communication and provision of information, and less commonly, the attitudes of HCWs, were causes of grievances. The likely causes of these issues are complex, and involve health system shortcomings such as nurse-patient ratios, doctor-patient communication and nurse-doctor relationships. Although difficult to address, the Singapore government continually plans to improve the system [36]. In this study, recommendations were not be targeted at a health system level, but have aimed to be as practical, simple and useful as possible for implementation on the ground.

This study also found that the healthcare-seeking behaviour of patients even in a developed country like Singapore, where healthcare is considered readily accessible and affordable, can be hindered by barriers like fear and inconvenience. There was also a general consensus that many more febrile patients were being screened for dengue since the introduction of the POCT kit. It is possible that this may have contributed to the increased incidence of dengue cases seen in recent years, however, further investigations will be necessary to confirm this finding.

### Strengths and limitations

To our knowledge, this is the first qualitative study done in Singapore exploring patient perceptions of health service delivery, as well as the first qualitative study exploring perceptions regarding usage of the Dengue Duo POCT kit. Our data went beyond analysis of diagnostics, taking a patient centred approach to understand where in the chain of care seeking delivery can be improved. Although these analyses were descriptive and synthesised using a comparative analysis, findings practically inform evidenced-based recommendations. We used a deductive framework [25] approach formulated for policy and practice lead analyses. All researchers came from an external organization and therefore participants did not feel hindered about sharing honest opinions. The analysis of data was conducted by TQ, who facilitated all FGDs and later interviews, providing more in-depth understanding of data.

There were also a number of limitations. Descriptive information about participants was not formally collected, and therefore our sample descriptors are limited to observable data. The credibility of FGD findings could have been affected by a non-response and recall bias, whilst respondents were perhaps those who had more free time (e.g. non-professionals, older or retired people), or had more opinions to express. Participants also could have recollected a lot more negative experiences than positive ones given the nature of the study [37]. Full data saturation was likely not reached in the patient groups as due to no-shows there were fewer than anticipated participants to conduct the focus group sessions with; although saturation was reached on broad themes in both patient and HCW analyses. Sampling of GPs occurred at a seminar on a Saturday during which many private clinics were open, so those who attended the seminar would have been public sector and particularly Dengue aware GPs.

## Conclusion

Health service delivery for dengue patients in Singapore is perceived to be of an acceptable clinical standard. However, patients expressed frustrations at many points in their patient journey, the major causes being waiting times and the lack of communication and information. HCWs felt that the hospital ED had the capacity to cope with the ‘dengue surge’ adequately, whilst the introduction of the POCT kit was viewed as helpful in improving the speed and accuracy of diagnoses. Improvements could be focused on the areas of care coordination between primary healthcare centres and hospitals, the enhancement of hospital service, and the effective delivery of information to patients. The AWARE model of care delivery can be considered for the improvement of hospital health service delivery for dengue patients.
